# Artificial Separations of the Teeth as a Means of Prevention of Decay

**Published:** 1876-06

**Authors:** G. R. Thomas


### SELECTED ARTICLES.

---

### ARTICLE III

### *Artificial Separations of the Teeth as a Means of Prevention of Decay.*

BY G. R. THOMAS.

**Read before the Michigan State Dental Society, March 29, 1876.**

For one to present before the Association an essay on general anatomy, I regard as of little value. The teaching of anatomy is a science and should be done only by those who have made a most thorough investigation of its beautiful mysteries. Instead of attempting the preparation of the subject assigned me, I have chosen to direct your attention to another, not less important, but more practical matter, and have secured the services of our much loved Prof. C. L. Ford, who has kindly consented to entertain and instruct us with one of his interesting talks on anatomy.

We, as dental surgeons, are called upon to operate daily upon the oral cavity, and principally upon the teeth. I do not believe that associations like this are places for the teaching of primitive principles, or the ground work of our profession, but rather for the exchange of modern views, the ventilation of new theories and the extirpation of false doctrines and dogmas The true man of to-day who is fitted for the practice of dentistry, is not thirsting so much for written pages upon anatomy, physiology, hygiene and their auxiliaries as for some practical means of saving the teeth of the children of this generation ; not so much for arresting as for preventing the decay. It is startling, gentlemen, but nevertheless true, that there are no American born children in this country to-day with perfect teeth, or, in other words, secure from the terrible malady that stands knocking at the gateway of the alimentary canal. The question is, what is to be done ?

" Tis true that without a pretty thorough knowledge of the anatomy of the human body, we, as curists, are unfit to take the first step in this great work. It is not only necessary for us to know the component parts of a tooth, but that it is a vascular, highly organized, and yet wholly dependent organ. We are all ready to admit, without question, that a tooth may remain serviceable for years that has been de. prived of all its internal nourishment, but is the answer as familiar to us as is the fact of its existence ? We know too that the teeth may be, and frequently are broken by accident, in some cases being deprived of one-third, or even one-half their crowns, and the balance remain untouched by decay for many years ; and I venture the assertion, that such teeth, if among the incisors or canines, are almost always free from future attacks of caries at the points of fracture, and this brings me to the beginning of what I wish to present in this paper.

The advisability of artificial, though permanent separation of the teeth of the children of the present generation, as a means of prevention of decay. But I fancy I hear some brethern in the advance guard saying, that assertion belongs to the history of the past. This is an advanced age, one of cohesive gold, mallet force and contour filling. True, but you must concede that there are times when the brakes must be applied, and that too before the impetus gained is such that certain destruction attend their application.

I expect to be met by opposition and shall be greatly discouraged if I am not. Hidden truth is never arrived at in any other way than by investigation.

Those of you who are familiar with Dr. Robert Arthur's work on "Treatment and prevention of decay of the teeth," will doubtless anticipate me in what I have to say.

What surfaces of the teeth are attacked by decay? The fissure, proximal, and rarely the labial (superior,) never any other, without it may be an extremely exceptional case. And what are the causes.

The object of this paper does not demand from me a presentation of the analysis of the many abnormal secretions of the buccal cavity, that doubtless play an active part in all the disintegrating processes.

The surfaces that I have mentioned, however, are affected by these secretions, for reasons that are very apparent The grinding, or fissure surfaces, partly because of their imperfections from the beginning, and partly from the readiness with which they retain the secretions that pass over them. Now if these surfaces were perfectly formed at the beginning and there were any means by which the secretions could be continually washed away, as they are by the saliva and tongue, they would no more suffer from decay, than do the lingual surfaces. Consequently the only known remedy as yet, by which these surfaces can be preserved, is to cut away and fill, and this may about as well be done before decay begins—if you can reach the child before such time—for it is sure to require being done sooner or later in almost every instance, *i. e.*, it is quite safe to say that the fissure surfaces that you have operated upon will in no wise offset the teeth that will be lost, from postponing the operation till decay is apparent, for in the one case you anticipate, while in the other it is usually left for the patient to detect, and you will know how little security there is in that.

It is claimed by Mr. Arthur, that if the child at an early age is placed entirely in the hands of the dentist, and he faithfully performs his duty, that none except the fissure surfaces need be allowed to decay.

Now I am not ready to accept this statement, without some modification. But I do believe it contains more truth than perhaps any of us are willing to concede. It is also claimed by Mr. Arthur, that the teeth, after having been thoroughly separated and polished, are no more liable to decay, than they would be, had their surfaces never been disturbed ; neither am I quite willing to accept this statement as entirely correct; but I am convinced from careful observation, that the teeth that have been thoroughly separated, one from the other, and caused to remain so, are much less liable to decay than those whose surfaces closely proximate, with all the enamel nature has seen fit to give them, undisturbed. Therefore, I make this statement, based upon my observation, that the few approximate surfaces that have never been disturbed, that we find in middle life have escaped decay, are no equivalent for the good that might have been accomplished, had all the proximate surfaces been separated in early life. If this is correct, then our duty is plain ; and so far as the separation of the temporary from the permanent teeth, as they appear, is concerned, I have not the slightest doubt. I believe in every instance, the temporary molars, should be separated from the six year molars, within six months after their eruption, leaving a good, large V shaped opening; and this course should be rigidly pursued with every temporary tooth, as fast as the neighbor permanent makes its appearance. The permanent teeth can in no wise suffer from this treatment, but on the contrary, must be benefitted. Granting for the sake of argument, that the temporary teeth are injured, it will be only for a few years at the longest. I think no one will question the correctness of this statement. That if a tooth occupies a position in the mouth alone, *i. e.*, well removed from any other tooth—as is frequently the case in abnormal eruptions—it is seldom attacked by decay on any other surface than the grinding, if a molar or bicuspid, and not at all if an incisor or cuspid.

This being true, then the main objection to this course of treatment will be upon this one point, that the unprotected dentine is more liable to decay than nature's polished enamel. I am aware that there are some other objections which I shall not stop to mention here, but shall expect them to be brought out, in the discussions that I hope may follow.

As a refutation of the objection named, I mention this fact. How many times have we all seen teeth that have been worn down upon their grinding surfaces, till they were but little above the gums, and how rarely is it that you see such teeth decayed upon these surfaces; almost never. What reason can be assigned? The enamel is gone, and has been for years; just this, a change takes place in the dentinal tubes, ossification of whatever substance fills those tubes, until we have what was once a cellular and highly vascular substance, changed to solid, resisting and nearly organic structure.

I am quite well aware of the fact, that upon suggesting such a course of treatment to a patient, the exclamation is almost universal.

"But does not that destroy the enamel?" They do not know as well as we, that in almost every instance of proximal fillings, where the decay has progressed to any great extent, more or less of the dentine is left exposed, when the filling is completed, and is it any the less liable to decay in the one case than the other? I claim that it is. I believe that in the case of a sound tooth where the enamel has been removed from its proximate surface, thoroughly polished, and remains a proper distance from its neighbor, that it is much less liable to decay than that tooth that has been found to be decayed on its proximal surface, filled, dressed down and polished in the usual manner, even if left a proper distance from its neighbor. It is not possible for the line of demarkation between gold and dentine, to remain as secure from future action of the secretions, as is highly polished dentine alone. I suppose if I was to make the assertion, that highly polished exposed dentine is better capable of resisting decay, than nature's own covering (enamel) I should make myself a subject of ridicule. But let us see if there is any ground for such belief. Why is it that the grinding surfaces of teeth that are worn away sometimes to the pulp itself are so free from decay ? Is it not because of a recuperative power, stimulated by the pulp, conveyed through the tubuli to the abraded surface ? I think there is no doubt about this. Is there any opportunity for this recuperative power to extend into and through the enamel ? None that I know of. In support of this belief I wish to quote from one of the ablest writers on the subject of comparative anatomy of the teeth.

Owen's Odontography, Introduction xxiii. In speaking of the structure of the enamel, he says : "Its highest condition of development is found in the mammalia," but further says "this condition of the enamel, however, like the corresponding one of the mammalian dentine, in the same degree as it distinguishes them from the true osseous tissues, and perfects them for their mechanical applications, removes them from the influence of the conservative and reparative powers of the living organism. The mammalian enamel therefore, once formed and exposed, is least able to resist vitally the influence of external decomposing forces. But this inferiority is amply compensated by its superior mechanical endowment."

There is no question about enamel being the best mechanical protector, but it will be observed that the point made by Owen is, that in proportion to the vitality of a part, is its power to resist the action of external decomposing agents. If this is true we all know the dentine is endowed with a power to resist the action of external agents, that the enamel in no degree possesses.

But gentlemen, I think a little unprejudiced observation by any man in the practice of dentistry will enable him to see that the truth of this theory is beyond question.

I have a lady in my care at the present time, whose teeth were put in order by one of the old school practitioners in Boston, 17 years ago ; when first coming to me for consultation, she complained of an uneasy feeling in the right cheek ; upon examination I found the posterior proximal surface of the first bicuspid, anterior surface of the second bicuspid, posterior surface of first molar, had all been filled with non-cohesive gold, and as near as I could judge by the appearance of the fillings, had all reached about the same degree of decay when filled. But for some reason the dentist had resorted to very different modes of procedure. In the case of the first and second bicuspids, he had chosen to cut away all, or nearly all, of what might be called the overhanging enamel, and then filling, thereby leaving a somewhat unsightly shaped opening, while in the other case he had adopted the other mode of ;ressing the teeth apart, filling, and allowing them to come together again. The result is that in the former case the teeth about the fillings to all appearance are just as perfect as when he left them, while in the latter, the decay has completely displaced the fillings, and progressed till the pulps in both were exposed the one of which I capped, the other I was obliged to ex tirpate. Now can there be any question, as to which of these two operations was the better ? Certainly none when we came to the result ; remember they were both in the same mouth, on the same side of the mouth, in the same jaw, of nearly the same size, placed there at the same time and by the same dentist, and this is only one of hundreds of cases, I believe I may safely say, that have come under my observation within the past ten years.

I hope I shall not be misunderstood in the statements I have made, and to show you that I do not believe in the wholesale slaughter that is sometimes made upon the proxi mal surfaces by ungainly separations I have prepared a series of separations upon this plaster model, which were obtained from an impression taken of this artificial set of teeth. By closely comparing the two you will see how little changed they appear, and yet you will easily discover how much more readily teeth like those upon the plaster cast would free themselves of all foreign substances, than would those like the ones on the plate; although this is by no means a fair exhibition of the principle, because the teeth in this case are so much smaller and shorter than natural ones, that the advantages to be derived do not fully appear. Had I not delayed the preparation of this paper to the very last, I should have been able to have produced an impression from the mouth, showing the case just as I have operated upon it.—*Dental Register.*

